# Engaging Youth in Blood Donation: A Call for Ethical Public Health Strategies

**DOI:** 10.21315/mjms-06-2025-453

**Published:** 2025-10-31

**Authors:** Yusrita Zolkefli

**Affiliations:** PAPRSB Institute of Health Sciences, Universiti Brunei Darussalam, Brunei Darussalam

Dear Editor,

The article “Knowledge and Attitude of Malaysian Public Towards Blood Donation During COVID-19 Pandemic: A Cross-Sectional Study” (1) brings attention to a critical public health issue in securing a consistent blood supply, especially during emergencies. A key takeaway from this research is the urgent need to reach out young people who might be uninformed, unsure, or reluctant about donating blood. For public health officials, overcoming this challenge is not merely a logistical task. It also demands an ethically sound approach. Inspiring young individuals to become regular donors requires strategies that respect their maturity level, protect their rights, and ensure fairness. This letter outlines how four core ethical principles, namely autonomy, beneficence, non-maleficence, and justice, can guide youth-focused blood donation initiatives.

The World Health Organisation reminds us that donating blood voluntarily is more than just a health measure. It also represents community solidarity and shared responsibility (2). Despite this, many nations still struggle with low donation rates due to limited awareness and misconceptions about the donation process (3). According to Abdullah Muzafar Shah et al. (1), the 18 to 25 age group holds significant potential as a stable donor base if effectively engaged. However, motivating this age group calls for more than simple awareness campaigns. It requires a respectful approach that allows them to make decisions freely and without undue pressure.

To begin with, respecting autonomy must be the cornerstone of any youth donor campaign. Adolescents and young adults should have access to trustworthy, clear, and age-appropriate information so they can make their own choices confidently. Blood donation should be portrayed as a meaningful option rather than a moral duty. Common fears, such as fear of needles or fainting, should be acknowledged and addressed openly. Clear communication and opportunities to ask questions help build trust and make young people feel safe and empowered. For example, in England, all secondary school students now receive education about blood donation as part of the standard curriculum. This initiative aims to get young people thinking about donation and discussing it with family members, thus boosting awareness and acceptance. Teachers have access to free, high-quality resources, and schools can host talks by guest speakers with real-life donation stories to inspire students (4). These efforts equip young people to make informed choices without feeling pressured.

The principles of beneficence and non-maleficence go hand in hand to ensure that young donors benefit while being protected from harm. It is vital to avoid campaigns that guilt-trip or manipulate young people into donating. Instead, supportive spaces that encourage honest conversations and peer encouragement help alleviate anxiety and build a positive donation experience. The Singapore Red Cross Youth Ambassador Programme is an excellent model whereby youth ambassadors promote blood donation within their social circles and communities while offering mental health support and opportunities for peer discussion after donation. This helps donors feel appreciated, cared for, and willing to donate again in the future (5). Likewise, the Australian Lifeblood Youth Programme invests in training staff and facilitators in youth psychology and communication skills. This ensures that young donors are approached with empathy and openness so their questions and fears are handled with care. This trust-building interaction increases the likelihood of repeat donations and creates long-term donor loyalty (6).

It is also crucial to recognise that younger donors are at a higher risk of experiencing specific adverse reactions, such as vasovagal reactions (7). This reinforces the role of blood services to minimise harm, for example, by conducting meticulous pre-donation screenings and establishing individualised donation intervals for younger donors within an ethical framework. Providing advice on hydration, rest, and dietary support both before and after the donation also contributes to the protection of their well-being. These practices maintain the principle of non-maleficence and promote trust, therefore ensuring the safety and sustainability of youth participation in blood donation.

Meanwhile, the principle of justice is equally vital for ensuring that blood donation opportunities are fair and accessible to all young people. This means breaking down barriers that prevent youth in rural areas, low-income households, or marginalised groups from participating. In Malaysia, for example, the National Blood Centre have set up mobile blood drives and outreach programmes that bring donation services directly to rural schools and remote communities. By working with local schools and community leaders, these efforts help bridge geographical and economic gaps and allow more young people to learn about blood donation and participate safely (8). Justice also means including those who cannot donate for health or personal reasons by giving them other ways to contribute, such as flexible donation hours, which can help those with transportation difficulties. Partnering with local communities is also crucial to maintain fairness and adapt to evolving needs.

Building on the principle of justice, engaging young people in blood donation not only extends this ethical value but also strengthens the healthcare system and promotes community health. Youth participation contributes to the resilience of the healthcare system and the promotion of public health from a broader perspective. Voluntary donation reinforces preparedness for health emergencies, fosters collective responsibility, and ensures equitable access to life-saving treatments. When combined with public health strategies, ethical frameworks ensure that youth-focused initiatives are both morally sound and socially sustainable. Table 1 summarises the integration of ethical principles with practical public health strategies to guide youth-focused blood donation initiatives, thereby illustrating this dual perspective.

In conclusion, the findings from this study (1) underscore the gaps in blood donation awareness and willingness among young Malaysians, especially during times of crisis. This mirrors global patterns and calls for ethical and sustainable approaches to involve youth meaningfully. Ensuring a stable blood supply means viewing young people not just as donors but as partners in public health. Respecting their autonomy, protecting their mental and physical well-being, recognising and managing the higher risk of adverse reactions among young donors, promoting fairness, and involving them actively builds lasting trust and a robust culture of voluntary donation. At the same time, situating blood donation within broader public health strategies highlights its role in strengthening community solidarity, health system resilience, and emergency preparedness. Eventually, the goal is to empower informed and compassionate young citizens who see blood donation as an act of solidarity and a meaningful contribution to collective health and one that they choose to make freely and safely.

## Figures and Tables

**Table 1:** Ethics and strategies in youth blood donation

Ethical principle	Youth-focused strategy	Public health outcome
Autonomy	Age-appropriate education, transparent communication	
Informed and voluntary participation	Beneficence Positive donation experiences, peer support programmes	Higher donor satisfaction and retention
Non-maleficence	Screening, pre- and post-donation support	Reduced adverse reactions, safer donor base
Justice	Mobile blood drives, rural outreach, and inclusive opportunities	Equitable access, stronger community health
